# Oral and Gut Health, (Neuro) Inflammation, and Central Sensitization in Chronic Pain: A Narrative Review of Mechanisms, Treatment Opportunities, and Research Agenda

**DOI:** 10.3390/ijms27010114

**Published:** 2025-12-22

**Authors:** Ishtiaq Ahmed, Jo Nijs, Matteo Vanroose, Doris Vandeputte, Sébastien Kindt, Ömer Elma, Jolien Hendrix, Eva Huysmans, Astrid Lahousse

**Affiliations:** 1 Pain in Motion Research Group, Department of Physical Therapy, Human Physiology and Anatomy, Faculty of Physical Education & Physical Therapy, Vrije Universiteit Brussel, Jette, 1090 Brussels, Belgium; matteo.vanroose@vub.be (M.V.); oelma@bournemouth.ac.uk (Ö.E.); jolien.hendrix@vub.be (J.H.); eva.huysmans@vub.be (E.H.); astrid.lucie.lahousse@vub.be (A.L.); 2 PijnPraxis.be Practice for Pain Management, 3970 Leopoldsburg, Belgium; 3 Unit of Physiotherapy, Department of Health and Rehabilitation, Institute of Neuroscience and Physiology, Sahlgrenska Academy, University of Gothenburg, SE-405 30 Gothenburg, Sweden; 4 Department of Clinical Sciences, Faculty of Medicine and Pharmacology, Vrije Universiteit Brussel, Jette, 1090 Brussels, Belgium; doris.vandeputte@vub.be; 5 Department of Gastroenterology and Hepatology, Universitair Ziekenhuis Brussel, Vrije Universiteit Brussel, Jette, 1090 Brussels, Belgium; sebastien.kindt@uzbrussel.be; 6 School of Allied Health and Exercise Sciences, Faculty of Health, Environment, and Medical Sciences, Bournemouth University, Bournemouth BH12 5BB, UK; 7 Department of Public Health and Primary Care, Centre for Environment & Health, KU Leuven, 3000 Leuven, Belgium; 8 Research Foundation—Flanders (FWO), 1000 Brussels, Belgium; 9 Department of Physical Medicine and Physiotherapy, Universitair Ziekenhuis Brussel, Jette, 1090 Brussels, Belgium

**Keywords:** gut–brain axis, dysbiosis, lifestyle medicine, back pain, neck pain, osteoarthritis, fibromyalgia

## Abstract

Given the limited efficacy of current interventions and the complexity of chronic pain, identifying perpetuating factors is crucial for uncovering new mechanistic pathways and treatment targets. The oral and gut microbiome has emerged as a potential modulator of pain through immune, metabolic, and neural mechanisms. Contemporary evidence indicates that chronic pain populations exhibit altered oral and gut microbiota, characterized by reduced short-chain fatty acid (SCFA)-producing taxa and an overrepresentation of pro-inflammatory species. These compositional changes affect metabolites such as SCFAs, bile acids, and microbial cell wall components, which interact with host receptors to promote peripheral and central sensitization. Microbiota-derived metabolites modulate peripheral sensitization by altering nociceptive neuron excitability and stimulating immune cells to release pro-inflammatory cytokines that increase blood–brain barrier permeability, activate microglia, and amplify neuroinflammation. Activated microglia further disrupt the balance between excitatory and inhibitory neurotransmission by enhancing glutamatergic activity and weakening GABAergic signaling, thereby contributing to the induction and maintenance of central sensitization. While observational studies establish associations between dysbiosis and chronic pain, animal models and early human fecal microbiota transplantation studies suggest a potential causal role of dysbiosis in pain, although human evidence remains preliminary and influenced by diet, lifestyle, and comorbidities. Overall, microbiota appears to regulate pain via peripheral and central mechanisms, and targeting it through specific interventions, such as dietary modulation to enhance SCFA production, alongside broader lifestyle measures like sleep, physical activity, stress management, and oral hygiene, may represent a new therapeutic strategy for the management of chronic pain.

## 1. Introduction

Chronic pain represents a major public health challenge. Low back and neck pain are the most common chronic pain conditions and rank among the leading causes of years lived with disability worldwide, with persistently high prevalence across regions and age groups [1,2]. In 2021, an estimated 629 million people experienced low back pain, and 253 million experienced neck pain [1], numbers projected to rise to 955 million and 281 million, respectively, by 2050 [1]. Low back and neck pain combined were the second leading cause of disability-adjusted life years (DALYs) globally in 2021, accounting for 90.6 million DALYs (95% UI, 63.8–123.0 million) [1]. Beyond personal suffering, chronic pain also imposes substantial economic costs, with direct healthcare expenditures and productivity losses in the United States alone estimated at $560–635 billion annually [3]. Current treatments, including nonpharmacological strategies such as cognitive behavioral therapy, exercise, and multidisciplinary care, provide meaningful benefits for many patients [4,5]. However, treatment effects are often modest, and a considerable proportion of individuals continue to experience persistent pain and functional limitations despite appropriate care [6,7,8]. This limited effectiveness may reflect the complex pathophysiology of chronic pain, which involves low-grade systemic inflammation [9,10] and central sensitization [11], defined as an amplification of neural signaling within the central nervous system that elicits pain hypersensitivity [11]. Given the limited effectiveness of current interventions and the complexity of chronic pain, identifying perpetuating factors is crucial for uncovering new mechanistic pathways and treatment targets.

Among emerging targets, the oral and gut microbiota have gained attention for their potential role in modulating pain through immune, metabolic, and neural pathways [12,13,14]. The oral and gut microbiota comprise diverse populations of microorganisms, including bacteria, fungi, protozoa, and viruses, inhabiting the oral cavity and gastrointestinal tract, respectively [15]. The gut microbiome further encompasses these organisms along with their genetic material, metabolites, and environmental interactions [13,16,17]. There is a significant overlap between oral and gut bacteria [18], with four phyla dominating both niches: Firmicutes, Bacteroidetes, Proteobacteria, and Actinobacteria, while Fusobacteria are also prominent in the oral microbiota [19,20,21]. Oral microbiota can translocate to the gut, and microbial communities in one niche are often predictive of the other, suggesting an integrated oral–gut axis [20]. Despite the anatomical continuity of the gastrointestinal tract and this integration between oral and gut microbial communities, most microbiome research in chronic pain has remained organ-specific, focusing mainly on the gut–brain axis and regarding the oral cavity as primarily relevant to local dental disease [22]. However, current evidence supports an integrated view, wherein the oral and gut microbiota together form a dynamic and interconnected system that influences metabolic functions, immune responses, and overall health [15]. This ecosystem is highly dynamic, shaped by intrinsic host factors such as genetics, sex, and intestinal physiology [16,23,24,25], and to an even larger extent by extrinsic influences including diet, geography, stress, physical activity, and sleep [16,17,25,26].

Alterations in gut microbiome composition, termed dysbiosis [16], can increase intestinal permeability [19], promote systemic inflammation [27], and have been linked to a wide range of diseases, including inflammatory [28], metabolic [29], neurological [30,31,32], and chronic pain conditions [13,14,27,32]. Emerging evidence suggests that the oral microbiome may contribute to chronic pain, thereby extending the microbiome–pain framework beyond the gut [33]. Oral dysbiosis has been associated with chronic pain [20,34,35]. Studies have reported poor oral health in different chronic pain populations [20], including those with fibromyalgia [36], abdominal pain [20], low back pain [37], and rheumatoid arthritis (RA) [38]. Recent advances in sequencing and metabolomics provide new opportunities to unravel the molecular mechanisms by which the oral and gut microbiome modulate systemic inflammation and drive peripheral and central sensitization through the oral–gut–brain axis.

Despite these advances and growing evidence supporting an integrated oral–gut axis, previous reviews have primarily focused on the gut–brain axis, with limited consideration of the oral microbiome. The aim of this review is to address this gap by synthesizing evidence on the integrated oral–gut–brain axis in chronic pain. It examines how microbial metabolites such as short-chain fatty acids (SCFAs), bile acids, and tryptophan derivatives are produced and how they modulate pain through peripheral and central sensitization mechanisms. It also highlights differences in microbial composition between healthy and chronic pain populations, considers emerging causal evidence, and discusses therapeutic strategies and future research directions.

### Search Strategy and Selection Criteria

Articles for this narrative review were identified by searching PubMed using the terms “gut microbiome”, “oral microbiome”, “dysbiosis”, “chronic pain” and “systemic health” up to August 2025. Articles were selected based on their relevance to the key arguments of this narrative review. Additional papers were identified from the reference lists of retrieved articles and through forward citation tracking of key publications. Only papers published in English were reviewed.

## 2. Microbial Metabolites

The oral cavity and gut lumen contain dense and diverse microbial communities that generate metabolites such as SCFAs, bioactive compounds, and secondary bile acids, which are fundamental to host physiology, immune regulation, and the development of disease [39]. Although the oral and gut niches are anatomically distinct, they interact through an oral–gut microbiota axis that jointly shapes the host’s metabolite and inflammatory milieu [40]. Oral microbes and their metabolites are continuously swallowed and can modulate gut microbial composition (dysbiosis) and epithelial barrier function, thereby shifting gut-derived metabolites (e.g., SCFAs, bile acids, and tryptophan metabolites) and other microbial-associated signals [20,40,41,42,43,44,45]. These combined oral- and gut-derived products may contribute to systemic immune activation and neuroimmune sensitization pathways relevant to nociception and central sensitization.

### 2.1. Metabolite Production by the Oral Microbiota

**Figure 1:**
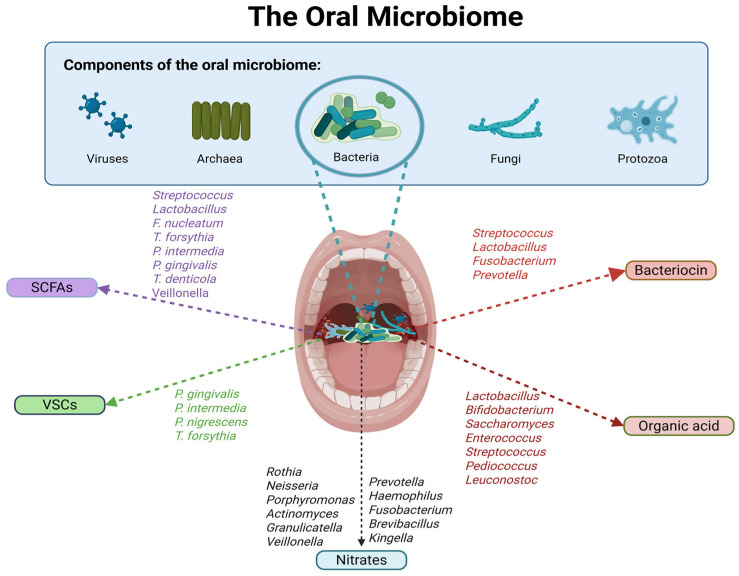
Oral microbiota genera and their major metabolic outputs (SCFAs, VSCs, nitrite/NO, bacteriocins) in the oral cavity, based on current evidence in the literature [47,51,52,53,54,55]. Abbreviations: SCFAs: short-chain fatty acids; VSCs: volatile sulfur compounds. Created with BioRender.com.

The oral cavity hosts more than 700 microbial species, making it the body’s second most diverse microbial community [46]. These communities generate diverse metabolites that shape oral health largely through immune regulation [33,47]. SCFAs in the oral cavity are generated mainly through two routes: carbohydrate fermentation and amino acid metabolism. Carbohydrates are fermented into monosaccharides and further converted to pyruvate via glycolysis or the pentose phosphate pathway [48]. Most oral bacteria produce SCFAs from pyruvate through the acetyl coenzyme A (acetyl-CoA) pathway, including *Streptococcus*, *Actinomyces*, *Lactobacillus*, *Propionibacterium*, *and Prevotella* [49]. Beyond dietary carbohydrates, salivary mucins rich in glycans can also serve as substrates once degraded by sialidases, supporting SCFA synthesis [50]. In addition, amino acids act as substrates for SCFA production, where proteolytic bacteria such as *Actinomyces*, *Veillonella*, and *Fusobacterium* degrade proteins and peptides, and subsequent deamination generates SCFAs [49]. Figure 1 shows the oral microbiota involved in the production of oral microbial metabolites.

### 2.2. Metabolite Production by the Gut Microbiota

The gut microbiota produce diverse metabolites by metabolizing dietary components, including SCFAs, secondary bile acids, vitamins, and amino acid-derived compounds such as indoles from tryptophan and phenols, which modulate host immunity, metabolism, nutrient homeostasis, and gut barrier integrity. Furthermore, bacterial cell wall components like lipopolysaccharides (LPS) from Gram-negative bacteria and peptidoglycans function as microbial-associated molecular patterns (MAMPs) that interact with host immune receptors, potentially leading to inflammation, endotoxemia, or systemic effects when intestinal permeability increases [56].

#### 2.2.1. Short-Chain Fatty Acids

**Figure 2:**
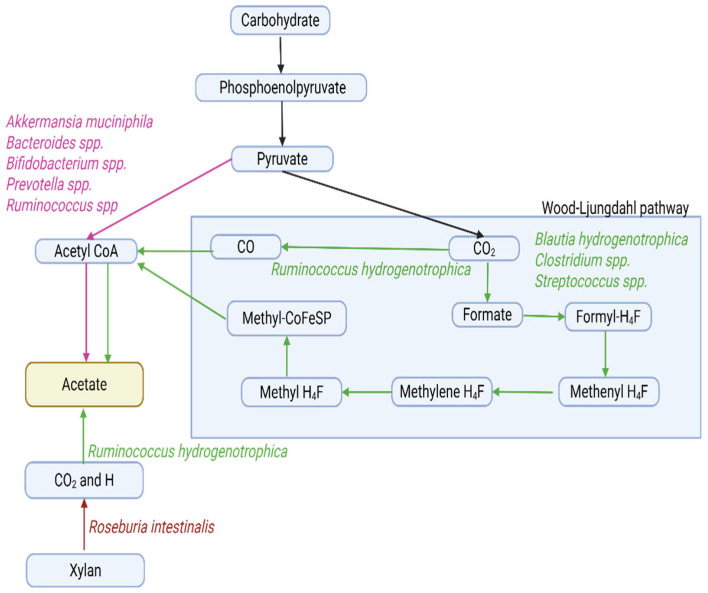
Microbial pathways for acetate biosynthesis from carbohydrates: Acetyl-CoA and Wood–Ljungdahl routes with key gut bacterial contributors. Bacterial species shown are based on published literature describing acetate-producing gut bacteria and are thus not exhaustive [57,61,62,63]. Carriage of the different pathways in gut microbes is indicated by color. Abbreviations: CoA, coenzyme A; CO_2_, Carbon dioxide; CO, Carbon monoxide; CoFeSP, Corrinoid iron–sulfur protein; H4F, tetrahydrofolate. Created with BioRender.com.

**Figure 3:**
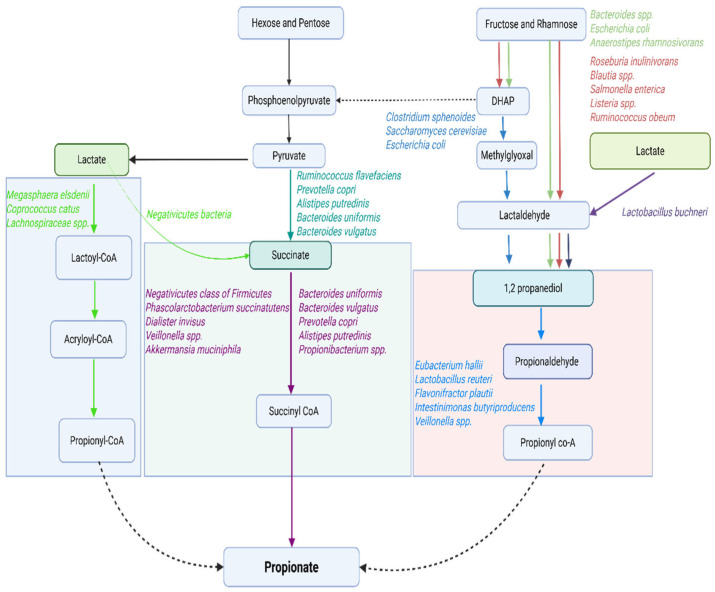
Microbial Pathways for Propionate Biosynthesis from Carbohydrates: Acrylate (Blue), Succinate (Green), and 1,2-Propanediol (Red) Routes with Key Bacterial Contributors. Bacterial species shown are based on published literature describing propionate-producing gut bacteria and are thus not exhaustive [56,57,61,62,63]. Carriage of the different pathways in gut microbes is indicated by color. Abbreviations: CoA, coenzyme A; DHAP, dihydroxyacetone phosphate. Dotted lines indicate that several intermediate steps are involved. Created with BioRender.com.

**Figure 4:**
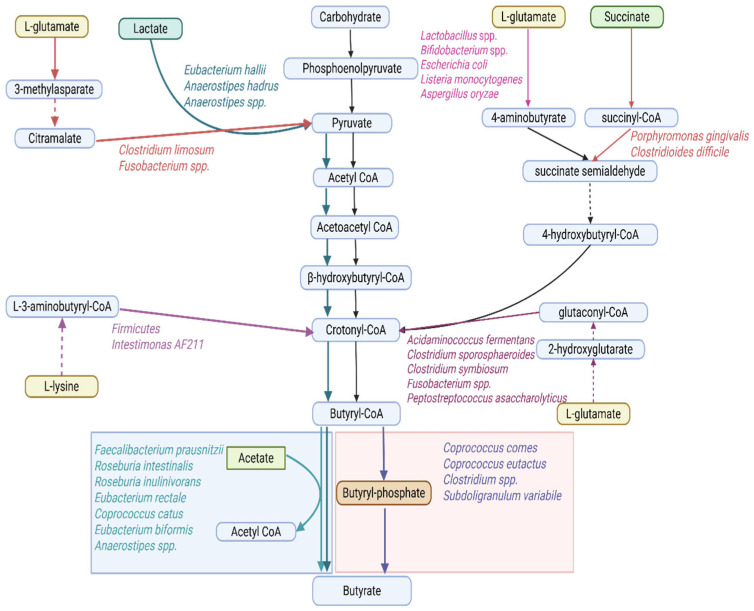
Microbial pathways for butyrate formation from carbohydrates, organic acids and amino acids. Butyryl CoA/acetate CoA-transferase (Blue) and phosphotransbutyrylase and butyrate kinase (Red) routes with Key Bacterial Contributors. Bacterial species shown are based on published literature describing butyrate-producing gut bacteria and are thus not exhaustive [56,57,61,62,63]. Carriage of the different pathways in gut microbes is indicated by color. Abbreviation: CoA, coenzyme A; spp, Species plural. Dotted line indicates that several intermediate steps are involved. Created with BioRender.com.

SCFAs are the most abundant microbial metabolites in the colonic lumen [17,57], mainly composed of acetate, propionate, and butyrate, with an approximate molar ratio of 60:20:20 [17]. They are produced through microbial fermentation of dietary fibers, with most metabolized by colonic epithelial cells and a smaller portion entering systemic circulation to influence host physiology [39]. Acetate is formed via the acetyl-CoA or Wood–Ljungdahl pathways [58,59] (Figure 2), propionate primarily through the succinate, acrylate, and propanediol routes [56,57,59] (Figure 3), and butyrate mainly through two routes, the butyryl CoA/acetate CoA transferase and the butyrate kinase pathways, using acetate as a co-substrate [57,59,60] or amino acids as alternative precursors [56] (Figure 4).

#### 2.2.2. Other Microbial Products: Amino Acid-Derived Metabolites, Bile Acids, and Structural Components

High protein intake promotes the microbial fermentation of undigested dietary proteins in the colon, producing amino acid-derived metabolites such as branched-chain fatty acids (isobutyrate, 2-methylbutyrate, isovalerate), phenylacetic acid, and other compounds, including phenols, indoles, p-cresol, amines, and ammonia, contributing minimally to the total SCFA pool [56,61,64,65]. *Bacteroides* spp. and certain *Firmicutes* degrade aromatic amino acids to yield these metabolites [65], which can exert both beneficial and detrimental effects on host metabolism, immunity, and inflammation [14,57]. Microbial tryptophan metabolism by *Clostridium sporogenes* and *Escherichia coli* produces indole, further converted by *Limosilactobacillus reuteri* (formerly *Lactobacillus reuteri*) and *Lactobacillus johnsonii* to indole-3-aldehyde [66,67]. Polyamines (putrescine, spermidine, spermine) arise from bacterial decarboxylation of ornithine and arginine via *Bifidobacterium* and *Lactobacillus* enzymes [61,68].

Primary bile acids escaping enterohepatic recirculation are deconjugated by bile salt hydrolases and converted via 7α-dehydroxylation by *Clostridium* (clusters XIVa and XI), *Eubacterium*, and *Bacteroides* into secondary bile acids such as deoxycholic and lithocholic acids, encoded in the *bai* operon [61,67,69,70,71]. In addition, microbial structural components such as LPS from Gram-negative bacteria [72,73] and peptidoglycan from both Gram-positive and Gram-negative taxa [67,74] serve as MAMPs detected by host pattern recognition receptors (PRRs), such as Toll-like receptors (TLRs) and nucleotide-binding oligomerization domain-like receptors (NLRs, including NOD1 and NOD2), potentially promoting low-grade inflammation and systemic immune activation when gut barrier function is impaired [75,76,77].

## 3. Mechanistic Pathways: Microbial Metabolites and Pain

### 3.1. Oral Dysbiosis and Pain Regulation

Periodontal and other oral dysbioses are increasingly recognized not only as local causes of gingival and periodontal tissue damage but also as initiators of systemic inflammatory and neuroimmune responses that extend far beyond the oral cavity. In healthy individuals, short-term cessation of oral hygiene led to increased gingival bleeding and elevated systemic inflammatory markers (high-sensitivity C-reactive protein (hsCRP), interleukin-6 (IL-6), and monocyte chemoattractant protein-1 (MCP-1)), which normalize upon resuming oral care [78]. Across systemically healthy periodontal disease and severe periodontitis, periodontal therapy modulates systemic inflammation: intensive treatment induces a transient inflammatory surge but subsequently improves endothelial function and reduces systemic inflammatory activity [79], while non-surgical therapy lowers circulating IL-6 (with only modest reductions in hsCRP) [80]. Consistent with these interventional findings, periodontitis is also associated with endothelial dysfunction and vascular inflammation, accompanied by elevated neutrophil counts and circulating IL-6 and C-reactive protein (CRP) [81]. At the cellular level, periodontal inflammation activates circulating monocytes through nuclear factor kappa-B (NF-κB) signaling, reinforcing systemic pro-inflammatory responses [82]. *Porphyromonas gingivalis* is found at low abundance in a subset of periodontally healthy individuals (reported in about 19% of healthy subjects and representing ~0.02% of the interdental biofilm) [83]. However, it acts as a keystone pathogen primarily within dysbiotic polymicrobial communities, where it orchestrates inflammation [84]. *Porphyromonas gingivalis* can directly influence host cells through the activation of NF-κB and mitogen-activated protein kinase (MAPK) pathways, thereby enhancing cytokine secretion and promoting low-grade inflammation [85]. Moreover, oral dysbiosis can induce production of pathogen-associated molecular patterns (PAMPs) signals, such as LPS, resulting in systemic stimulation of innate immune responses and inflammatory transcription factors, including NF-κB [86].

Beyond these systemic effects, oral dysbiosis characteristic of periodontitis also triggers neuroinflammatory responses. Recent studies have found associations between several oral microbial taxa and RA [35,87], fibromyalgia [20,34], and migraine [20]. However, it is important to emphasize that current evidence linking oral dysbiosis to chronic pain is predominantly cross-sectional and associative; therefore, it remains unclear whether oral dysbiosis causally contributes to systemic inflammation, immune priming, or central sensitization in chronic pain, or instead reflects comorbidities and shared risk factors. *Fusobacterium nucleatum* infection promotes microglial proliferation and activation via its lipopolysaccharide component, increases brain tumor necrosis factor-alpha (TNF-α) and IL-1β expression, and upregulates myeloid differentiation primary response 88 (MyD88), phosphorylated p38 (p-p38), and c-Jun N-terminal kinase (JNK) signaling, indicating localized microglial-mediated neuroinflammation following oral infection [88]. *Porphyromonas gingivalis* produces gingipains that citrullinate host proteins, trigger TLR2/4 activation, and promote local and systemic inflammation [87]. Accordingly, *Porphyromonas gingivalis* and its gingipain proteases were detected in brain tissue and cerebrospinal fluid of Alzheimer’s disease patients, where their presence correlated with neuroinflammatory signaling [89]. In mice, chronic oral infection resulted in hippocampal colonization by *Porphyromonas gingivalis*/gingipain, microgliosis, astrogliosis, and marked elevation of IL-1β, IL-6, and TNFα expression, accompanied by neuronal loss [89,90,91]. Moreover, *Porphyromonas gingivalis* and its outer membrane vesicles can activate NLRP3 and NLRP1 inflammasomes, triggering pyroptotic cell death and release of neuroinflammatory interleukins (IL-1β and IL-18), processes that may further amplify neuroinflammation [89,92]. Pharmacological inhibition of gingipains reduced bacterial burden and suppressed hippocampal TNFα mRNA, confirming attenuation of neuroinflammation [89]. Complementary evidence from periodontal ligature models shows increased IL-1β, IL-6, IL-8, and IL-21 in both peripheral blood and brain, with cortical and hippocampal microglial activation and engagement of the TLR/NF-κB-Signal Transducer and Activator of Transcription 3 (STAT3) pathway, linking systemic inflammation with neuroinflammatory responses [93].

Experimental models demonstrate that oral bacterial components and chronic inflammation alter trigeminal nociceptive processing. *Porphyromonas gingivalis*-driven infection modifies mechanical nociceptive thresholds [94]. *Porphyromonas gingivalis* lipopolysaccharide activates trigeminal sensory neurons via TLR4-dependent mechanisms, triggering calcitonin gene-related peptide release and NF-κB nuclear translocation, thereby modulating neuronal excitability and potentially contributing to acute and chronic inflammatory pain [95]. In a rat model of periapical periodontitis, pulpal inflammation enhanced neuronal activity in the trigeminal subnucleus interpolaris/caudalis (Vi/Vc) region, increasing Fos and phosphorylated extracellular signal-regulated kinase (ERK) expression and facilitating masseter hyperalgesia, suggesting that Vi/Vc and Vc nociceptive neurons contribute to orofacial pain hypersensitivity associated with dental inflammation [96]. Consistently, chronic inflammation of the tooth pulp induces bilateral and sustained phosphorylation of ERK and p38 MAPK in Vc, suggesting sustained intracellular signaling changes within neurons and astrocytes that underpin chronic pulpitic pain [97].

### 3.2. Oral–Gut Microbiota Axis in Pain Regulation

The oral cavity and gut contain dense and diverse microbial communities that interact through enteric, hematogenous, and immune pathways, collectively shaping the oral–gut microbiota axis [40]. Recent studies suggest that the oral–gut microbiota axis may serve as a potential causal link between oral health and systemic disease [40,46,98,99,100].

#### 3.2.1. Enteric Pathway

Oral microbes can translocate to the distal gut via the enteric pathway through daily saliva swallowing, with humans producing approximately 1 to 1.5 L of saliva containing around 1.5 × 10^12^ bacteria per day [101]. This pathway allows acid-resistant oral taxa, such as *Porphyromonas gingivalis*, *Helicobacter pylori*, *Fusobacterium nucleatum*, *Klebsiella*, and *Streptococcus* spp., to withstand gastric passage and colonize the intestine, contributing to gut dysbiosis [40,102,103]. Such enteric translocation of oral microbiota can alter intestinal microbiota (dysbiosis) and compromise epithelial barrier integrity, initiating systemic inflammation and neuroimmune activation [40,41,42,43,44,45]. Chronic periodontal dysbiosis, particularly driven by *Porphyromonas gingivalis*, activates the TLR/NF-κB signaling cascade in intestinal and neural tissues, amplifying cytokine release and sustaining inflammatory communication along the oral–gut–brain axis [46]. In rodents, oral *Fusobacterium nucleatum* exposure exacerbates visceral hypersensitivity alongside gut dysbiosis, supporting a gut-mediated contribution to peripheral nociceptive sensitization [104]. Pro-inflammatory mediators such as IL-1β, IL-6, and TNF-α, together with microglial activation, are well-established facilitators to peripheral and central sensitization [105,106]. Oral *Porphyromonas gingivalis* exposure, for example, has been shown to disrupt gut barrier integrity through decreased intestinal zonula occludens-1 (ZO-1) expression, increase colonic TNF-α and IL-1β, elevate serum IL-17A and brain IL-17 receptor A, and activate microglia in the substantia nigra [107,108]. Collectively, these findings indicate that oral microbiota–induced alterations along the oral–gut axis can drive gut barrier dysfunction, systemic cytokine signaling, and neuroinflammation, which are mechanistically consistent with processes that facilitate peripheral and central sensitization.

#### 3.2.2. Hematogenous Pathway

Oral bacteria can enter the bloodstream during routine activities such as toothbrushing and chewing or following dental procedures, with greater frequency and magnitude in individuals with periodontitis [40]. These episodes of transient bacteremia enable oral pathogens and their byproducts to disseminate systemically, where they can trigger endotoxemia, activate immune cells, and drive cytokine-mediated inflammation [82,109]. For example, *Fusobacterium nucleatum*-induced apical periodontitis in rats caused bacterial dissemination beyond the oral cavity, with *Fusobacterium nucleatum* DNA detected in the gut [110]. Histopathological analyses confirmed inflammatory cell infiltration in periapical tissues, while 16S rRNA sequencing revealed altered microbial composition across the gut, heart, liver, and kidney. These findings indicate that oral infection with *F. nucleatum* can reshape gut microbial communities and promote systemic dysbiosis through hematogenous dissemination [110]. Similarly, translocation of *Porphyromonas gingivalis* into the bloodstream increases cytokine production and drives mononuclear cell differentiation into highly active osteoclasts, contributing to inflammatory bone loss disorders such as RA [111].

#### 3.2.3. Immune Pathway

**Figure 5:**
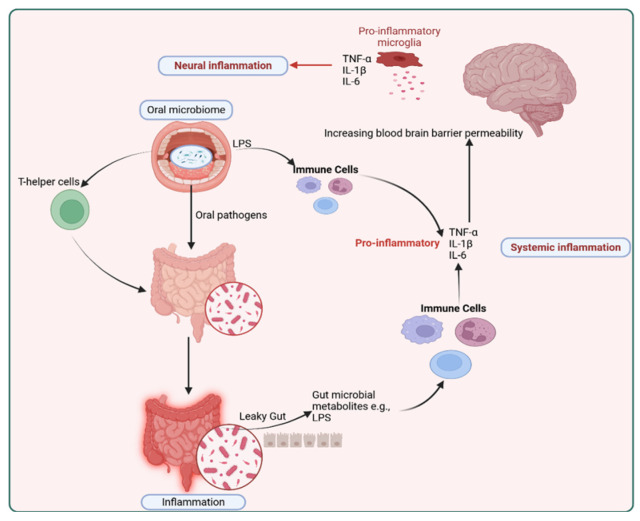
The oral microbiome influences systemic inflammation through the oral–gut axis via various microbial metabolites and specific bacterial taxa. Abbreviations: LPS, lipopolysaccharides; TNF-α, tumor necrosis factor-alpha; IL-1β, interleukin-1 beta; IL-6, interleukin-6. Created with BioRender.com.

Pathogenic microorganisms influence systemic inflammation through interconnected immune pathways in both the oral cavity and the gut. Certain oral pathogens, such as *Porphyromonas gingivalis* and *Fusobacterium nucleatum*, invade and colonize the oral epithelial cells and periodontal tissues, where the bacteria can release virulence factors and toxins that disrupt the integrity of the oral mucosa [112]. This disruption permits the oral microbes to penetrate deeper into gingival tissue. These microbes then interact with local immune cells to trigger activation and recruitment of neutrophils and natural killer cells, subsequently enabling dendritic cells to prime CD4+ and CD8+ T cells, thereby promoting inflammation through cytokine secretion [113]. Orally primed T cells can migrate to the intestinal mucosa, where they aggravate barrier dysfunction and inflammation [40] (Figure 5).

### 3.3. Microbiota–Gut–Brain Axis in Pain Regulation

The microbiota–gut–brain axis represents a bidirectional communication system through which gut microbiota influence the central nervous system via immune, autonomic, and neuroendocrine pathways [14,17,19,114]. Dysregulation of these interactions has been increasingly linked to neuroinflammatory processes that precipitate central sensitization [14], a maladaptive amplification of nociceptive signaling underlying many chronic pain conditions [11].

#### 3.3.1. Immunoregulatory Pathways

Gut microbiota influence pain through multiple immunoregulatory mechanisms, shaping peripheral and central sensitization through converging systemic and neuroinflammatory processes. The gut microbiome interacts with immune cells to modulate cytokine levels and prostaglandin signaling to influence brain function [17,115]. Experimental evidence indicates that the absence of commensal gut microbiota disrupts neuroimmune homeostasis and alters structural integrity within pain-regulatory circuits, as evidenced by heightened visceral sensitivity, increased spinal TLR and cytokine (IL-6, TNF-α, IL-1α/β, IL-10) expression, glial activation, and morphological alterations in pain-related brain regions (reduced anterior cingulate cortex volume, enlarged periaqueductal gray, and dendritic or spine hypertrophy). Restoration of the microbiota normalized these changes, underscoring its essential role in maintaining balanced neuroimmune and pain-processing mechanisms [116]. Similarly, dysbiosis is associated with increased production of pro-inflammatory cytokines such as IL-1β and TNF-α, which can cross the blood–brain barrier and activate microglia, sustaining neuroinflammatory cascades and facilitating central sensitization [117,118]. Following peripheral nerve injury, dysbiosis exacerbates pain through elevated spinal TNF-α, IL-1β, and glial activation, facilitating central pain sensitization, whereas probiotic administration suppressed these inflammatory and nociceptive changes [119]. Microbiota-derived metabolites, such as SCFAs, bile acids, and tryptophan derivatives, can act on host receptors (e.g., G-protein-coupled receptor (GPR) 43, TLRs, and transient receptor potential (TRP) channels), thereby modifying immune and neuronal signaling in ways that influence nociceptive plasticity (explained in Section 3.4) [14,27]. Translational evidence shows that microbiota from patients with fibromyalgia can transfer mechanical hypersensitivity and immune alterations to germ-free mice, whereas fecal microbiota transplantation from healthy donors alleviates symptoms [120]. Collectively, these data demonstrate that gut microbial communities regulate pain through systemic cytokine induction, microglial priming, and metabolite-mediated immune signaling.

#### 3.3.2. Autonomic Pathways

The autonomic nervous system forms an essential link between the gut and the brain, with the vagus nerve and the enteric nervous system playing central roles. Microbial metabolites and commensal signals influence brain and pain pathways through vagal afferents projecting from the gut to brainstem nuclei, where serotonergic, opioid, and cholinergic anti-inflammatory circuits modulate nociception, stress responses, and systemic inflammation [17,27,121,122,123,124,125]. By regulating motility and secretion, the vagus nerve supports gut barrier integrity and microbial balance, controlling systemic inflammation and maintaining immune homeostasis [27].

Experimental evidence demonstrates that specific microbial taxa directly modulate autonomic and sensory activity. In rats, oral administration of *Lactobacillus reuteri* inhibited the cardio-autonomic reflex and prevented the increase in colonic dorsal root ganglion excitability during colorectal distension, indicating that microbial signals can attenuate visceral pain through local enteric–autonomic mechanisms [126,127]. Similarly, *Lactobacillus rhamnosus* increased vagal afferent firing and responsiveness to gut distension and suppressed visceral pain responses to colorectal distension, demonstrating that specific microbes can modulate both acute vagal signaling and longer-term visceral nociception through integrated enteric and autonomic pathways [128]. The gut microbiota also modulate vagal afferent activity through metabolites such as bile acids, short-chain fatty acids, and 3-indoxyl sulfate, which act via G protein-coupled bile acid receptor 1 (GPBAR1), GPR43, and TRP ankyrin 1 (TRPA1) receptors. These findings show that microbial metabolites serve as chemical mediators of gut–brain communication, linking gut signals to autonomic and brain function [129]. In the absence of commensal microbiota, enteric sensory neurons display reduced excitability and altered membrane properties, which normalize following microbial colonization, underscoring the essential role of gut microbes in maintaining enteric neuronal and sensory function [130].

Microbiota–vagus nerve interactions are further supported by evidence from microbial transfer studies. Transplantation of stress-altered gut microbiota to healthy mice activated vagal pathways and disrupted serotonin–dopamine signaling, inducing hippocampal neuroinflammation and reduced neurogenesis; these effects were abolished by vagotomy, confirming that vagal integrity is essential for microbiota-driven modulation of brain and behavioral responses [131].

#### 3.3.3. Neuroendocrine Pathways

The hypothalamic–pituitary–adrenal (HPA) axis regulates stress responses and modulates the gut–brain axis through corticotropin-releasing hormone (CRH)- and adrenocorticotropic hormone (ACTH)-driven cortisol release [132,133,134]. In people with chronic pain, the HPA axis is often dysfunctional, implying alteration of corticosteroid expression, which can imply two opposite phenomena, namely hyper- and hypo-cortisolism [135]. Hypercortisolism is characterized by basal hypercortisolism and/or hyperreactivity, with basal hypercortisolism defined as a permanently increased cortisol level and decreased HPA axis negative feedback system, whereas hyperreactivity refers to normal cortisol levels with exaggerated behavioral and cortisol responses to stressful events [136]. Hypercortisolism has been found in several chronic pain conditions, including myofascial pain and burning mouth syndrome [137,138], and hypocortisolism in patients with myalgic encephalomyelitis/chronic fatigue syndrome (ME/CFS), irritable bowel syndrome, fibromyalgia and chronic pelvic pain [139,140,141,142]. While acute cortisol effects imply strong anti-inflammatory properties, chronic elevation of cortisol alters microbiota [27], increases gut permeability [143], and promotes neuroinflammation [144]. Chronic high cortisol is also linked to hippocampal atrophy, impaired stress regulation, and heightened nociceptive sensitivity [27,145]. In addition, gut microbes modulate neurotransmitters such as gamma-aminobutyric acid (GABA), serotonin, and melatonin, which influence nociceptive signaling, mood, and sleep [27,121].

Together, the immune, autonomic, and neuroendocrine pathways provide the major routes by which microbial activity in the gut influences nociceptive processing [14,118]. Their actions on peripheral and central sensitization are discussed in the following sections.

### 3.4. Microbial Metabolites Modulating Peripheral Sensitization

Microbiota-derived mediators influence dorsal root ganglia neurons both directly, by acting on receptors and ion channels (TLRs, TRPs, GABA), and indirectly, by stimulating immune and other non-neuronal cells to release cytokines (e.g., TNF-α, IL-1β, and IL-6) or chemokines (e.g., C–C motif chemokine ligand 2 (CCL2) and C–X–C motif chemokine ligand 1 (CXCL1)) [13,14,39,146].

#### 3.4.1. Short-Chain Fatty Acids

SCFAs primarily exert anti-inflammatory effects by modulating key signaling hubs like NF-kB, MAPK, and mammalian target of rapamycin (mTOR). These effects occur through GPR binding or histone deacetylase (HDAC) inhibition following cellular entry via transporters (e.g., Na^+^-coupled SMCT1/2 on the apical epithelium and H^+^-coupled MCT1/4 on both apical and basolateral membranes) or passive diffusion [13,39]. However, these actions are context-dependent, with paradoxical pro-inflammatory outcomes in pathological states. For example, acetate can engage GPR43 on neutrophils, potentiating their chemotaxis, oxidative burst, and cytokine production [14]. Their actions depend on carbon chain length, with acetate favoring GPR43, propionate targeting GPR41 and GPR43, and butyrate preferentially binding GPR109A and to some extent GPR41 [39,147]. Activation of GPR109A inhibits TLR4-induced expression and secretion of TNFα, IL-6 and CCl-2 and activation of GPR109A by butyrate exerts anti-inflammatory effects in colonic inflammation [148].

Microbiota-derived SCFAs can also modulate GPR41-mediated primary nociceptor excitability at the trigeminal ganglion, thereby shaping peripheral input to the spinal trigeminal nucleus caudalis (SpVc) [149]. In vivo studies demonstrate that intravenous propionic acid rapidly and reversibly suppresses SpVc wide-dynamic-range neuronal firing in response to mechanical stimuli [149]. In models of inflammatory hyperalgesia, butyrate reduces inflammatory hyperexcitability in nociceptive primary trigeminal ganglion neurons, thereby alleviating inflammatory hyperalgesia [150]. Evidence suggests that activation of satellite glia in sensory ganglia may also play an important role in the development of hyperalgesia and allodynia [151]. GPR43/GPR109A was detected in satellite glial cells of the dorsal root ganglia in the peripheral nervous system [152,153], implying that SCFAs could modulate satellite glial cells and thereby shape the local cytokine/chemokine milieu within ganglia.

##### NF-κB Pathway

NF-kB mediates the transcription of various cytokines (such as the cytokines TNF-a, TNF-b, IL-1β, IL-2, IL-3, IL-5, IL-12, and IL-18) and chemokines (IL-8, MIP-1a, CXCL-2, and CCL-2) [39]. Two subunits of NF-kB, P65 and P50, are acetylated and transferred from the cytoplasm into the nucleus to promote the secretion of pro-inflammatory cytokines [154]. SCFAs suppress NF-κB primarily through HDAC inhibition and GPR signaling [14,39,147]. Upon GPR43 binding, SCFAs recruit Gq/11 subunits to activate phospholipase C, generating inositol trisphosphate (IP3) and diacylglycerol (DAG), which trigger endoplasmic reticulum Ca^2+^ release [14]. This elevates intracellular calcium, activating protein kinase A (PKA) to ubiquitinate the NLRP3 inflammasome for autophagic degradation, thereby inhibiting NF-κB-driven cytokine release [14]. In experimental models of diabetic nephropathy, butyrate acts through GPR43-β-arrestin-2 signaling, enhancing the interaction between β-arrestin-2 and IκBα. This stabilizes IκBα, prevents NF-κB nuclear translocation, and reduces oxidative stress, collectively suppressing inflammatory responses [155,156].

**Figure 6:**
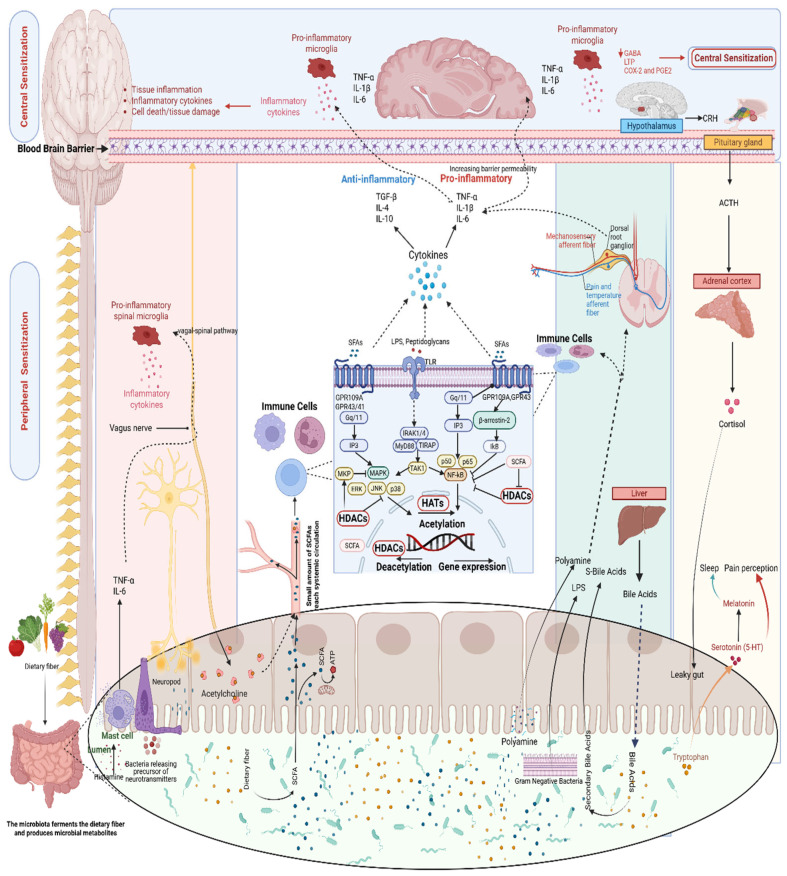
Schematic overview of gut microbiota-derived metabolites regulating peripheral and central pain sensitization through neural, immune, and endocrine pathways. Created with BioRender.com. At the bottom of Figure 6, microbial fermentation produces microbial metabolites like SCFAs, BAs, and neurotransmitters (e.g., serotonin, GABA), while structural components of microbes provide PAMPs such as LPS and peptidoglycans. On the left (neural pathway), vagal and spinal pathways convey microbial signals, with epithelial mediators such as serotonin from enterochromaffin cells and acetylcholine from neuropods, together with cytokines, directly modulating dorsal root ganglion excitability via different receptors. Mast cell-derived histamine also activates vagal/spinal pathways, stimulating spinal microglia to release pro-inflammatory cytokines, thereby bridging peripheral inputs and central sensitization. In the center (immune pathway), LPS and other microbial products activate immune cells, inducing TNF-α, IL-1β, and IL-6 that amplify nociceptive signaling. Anti-inflammatory regulation occurs through SCFAs acting on GPR43/41/109A and inhibiting HDACs, as well as kynurenic acid acting on GPR35. On the right (endocrine pathway), chronic pain results in a dysfunctional HPA axis with hyper- or hypocortisolism. Acute cortisol exerts anti-inflammatory effects, whereas chronic dysregulation promotes gut permeability. This promotes LPS translocation and alters bile acid and polyamine metabolism, which act on immune cells to drive pro-inflammatory cytokine release. In parallel, tryptophan metabolism is shifted toward serotonin and melatonin pathways, linking endocrine signaling with immune responses, sleep regulation, and nociceptive sensitivity. At the top (central sensitization), pro-inflammatory cytokines disrupt the blood–brain barrier, activating microglia to release glutamate, ATP, and PGE2, enhancing NMDA receptor phosphorylation, reducing GABAergic tone, and sustaining neuroinflammation and nociceptive hypersensitivity.

Additionally, HDAC inhibition by SCFAs deacetylates NF-κB subunits p65 and p50, enhancing p65-IκBα binding to export the complex from the nucleus and curb pro-inflammatory transcription [39,157]. Butyrate (80% inhibitory efficiency) and propionate (60% inhibitory efficiency) are known HDAC inhibitors that regulate NF-kB activity. They upregulate the production of IL-10 and inhibit the production of the pro-inflammatory molecules, including IL-12, TNF-a, IL-1β, and nitric oxide (NO) [39,158]. In human colonic epithelial cell models, HDAC inhibition by butyrate suppresses proteasome activity by downregulating the catalytic β-subunits (β1, β2, and β5), thereby preventing the proteasome-dependent degradation of IκBα and blocking TNF-α-induced NF-κB activation, while sparing proteasome-independent signaling. This selective interference with the proteasome-dependent pathway contributes to butyrate’s anti-inflammatory and anti-neoplastic actions [159]. Butyrate reduced TNF production and pro-inflammatory cytokine mRNA expression in intestinal biopsies and lamina propria mononuclear cells from Crohn’s disease patients. It inhibited LPS-induced cytokine expression and NFκB nuclear translocation in peripheral blood mononuclear cells, decreased NFκB transcriptional activity while maintaining IκBα stability, and ameliorated trinitrobenzene sulfonic acid-induced colitis, indicating suppression of inflammation through NFκB inhibition [160] (Figure 6).

##### MAPK Pathway

SCFAs modulate the MAPK family (ERK, JNK, p38), which controls gene transcription and pro-inflammatory cytokine secretion, through a balance of inhibition and activation [39]. SCFAs inhibit HDACs to acetylate and stabilize MAPK phosphatase-1 (MKP-1), enabling dephosphorylation of ERK/JNK/p38 and reducing downstream inflammatory signals [133,161]. However, activation of GPR41/43 by acetate can promote pro-inflammatory effects via activation of the extracellular signal-regulated kinases 1/2 (ERK1/2) (via both receptors) and p38 MAPK (via GPR43) signaling pathways, thereby increasing the production of cytokines (IL-6 and CXCL1/2) and chemokines like CXCL-1α and CXCL-2 [148] (Figure 6). This paradoxical pro-inflammatory potential highlights the complexity of targeting SCFAs therapeutically, as SCFAs may exert context-dependent pro-inflammatory effects via GPR43-mediated immune cell activation in specific pathological settings.

##### mTOR Pathway

SCFAs target the mTOR pathway, encompassing mTORC1/2 complexes that oversee cell growth, transcription, barrier function in the gut, and cytokine regulation, mainly through metabolic reprogramming [39]. mTOR increases acetyl-CoA content via the glycolysis pathway, and acetyl-CoA in the nucleus promotes the binding of acetyl groups to histones, thereby increasing the acetylation of histones and ultimately regulating gene expression and the production of cytokines such as IL-10 and TNF [39]. SCFAs inhibit HDAC and increase the acetylation of p70 S6 kinase and the phosphorylation of rS6, thereby regulating the mTOR pathway [162]. This HDAC inhibition by SCFAs shifts the balance toward enhanced histone acetylation, promoting anti-inflammatory effects through elevated IL-10 and modulation of pro-inflammatory cytokines [39] (Figure 6).

#### 3.4.2. Amino Acid Fermentation and Bioactive Microbial Metabolites

Gut microbes metabolize dietary amino acids such as tryptophan and histidine into a range of bioactive compounds that influence pain signaling. Tryptophan metabolites include indoles, serotonin, and kynurenic acid. Indoles can activate TLR4 and TRPA1 channels on dorsal root ganglion neurons, leading to calcium influx, oxidative stress, and neuropeptide release, which in turn increase excitability and promote neurogenic inflammation [14]. Indoles also stimulate the aryl hydrocarbon receptor (AhR), which promotes the release of IL-6 and TNF-a upon activation, enhancing pro-inflammatory IL-17 signaling [163] and amplifying neuroinflammation and nociceptor hyperexcitability. In gut epithelial cells, AhR activation also increases serotonin release, which sensitizes TRPV1 channels on afferent neurons, heightening visceral hypersensitivity. Indoles can also directly activate chloride channels by binding to the extracellular domain of the GABA-A receptor and induce hyperpolarization of the resting membrane potential in dorsal root ganglion neurons, thereby reducing the frequency of action potential firing [14].

Polyamines, including putrescine, spermidine, and spermine, modulate neuronal excitability by influencing N-methyl-D-aspartate (NMDA) receptor activity and microglial activation [164]. Dysregulated polyamine metabolism in dysbiosis promotes neuroinflammation and enhances glutamatergic signaling [146], thereby contributing to central sensitization [165].

#### 3.4.3. Bile Acid

Secondary bile acids such as deoxycholic acid and lithocholic acid modulate nociception through both pronociceptive and antinociceptive mechanisms. On the pronociceptive side, activation of TLR4 on dorsal root ganglion neurons upregulates TRPV4 channel expression via NF-κB signaling, sensitizing the channels and promoting calcium influx and neuronal hyperexcitability [14]. In addition, it activates ERK1/2 pathway via TLR4, enhancing adenosine triphosphate (ATP)-induced calcium inward flow and synergistically amplifying neuronal hyperexcitability [166]. Bile acids also disrupt inhibitory control by inducing endocytosis of GABA-A receptors, which reduces chloride currents and promotes depolarization [14]. Conversely, bile acids produce antinociceptive effect by acting on GPBAR1, expressed on sensory neurons and macrophages, where receptor activation triggers TRPA1-dependent calcium influx and itch responses in neurons but promotes analgesia through opioid release in macrophages. Beyond bile acids, the microbiota contributes to the production of kynurenic acid, which signals through GPR35 in dorsal root ganglion neurons to reduce excitability and induce dose-dependent analgesia in vivo [14].

#### 3.4.4. Microbial Cell Wall-Derived Metabolites

Microbial cell wall components, including LPS, peptidoglycans, and β-glucans, act as PAMPs of gut origin that are highly relevant to chronic pain [14]. Once released into circulation, they activate TLRs on immune cells and sensory neurons in the dorsal root ganglia, inducing innate immune activation and neuroinflammation that promote peripheral sensitization in models of neuropathic and inflammatory pain [12]. In neuroinflammatory conditions, they promote glial activation and cytokine release, amplifying peripheral nerve hyperexcitability [14,67]. LPS binds TLR4 on peripheral macrophages and dorsal root ganglia neurons, initiating NF-κB signaling that upregulates pro-inflammatory cytokines (e.g., TNF-α, IL-1β), resulting in peripheral sensitization [12,14]. Lipoproteins and peptidoglycans activate TLR2 on peripheral immune cells and sensory neurons, initiating downstream signaling [14]. This activation recruits Toll/IL-1 receptor domain-containing adaptor protein (TIRAP) and MyD88. Together, these adaptor proteins assemble a complex that phosphorylates interleukin-1 receptor-associated kinases (IRAK1 and IRAK4). The activated kinases interact with tumor necrosis factor receptor-associated factor 6 (TRAF6), forming a signaling hub that stimulates transforming growth factor β-activated kinase 1 (TAK1). TAK1 then activates both NF-κB and MAPK pathways. NF-κB activation promotes transcription of pro-inflammatory cytokines such as TNF-α, IL-6, and IL-1β, which promote the activation of macrophages, dendritic cells, and B lymphocytes. These immune cells, once activated, further amplify inflammation through sustained release of TNF-α, IL-12, and interferon gamma (IFN-γ), reinforcing inflammatory signaling via autocrine and paracrine loops [14] (Figure 6).

### 3.5. Microbial Metabolites Modulating Central Sensitization

Under neuroinflammatory conditions, pro-inflammatory cytokines such as TNF-α, IL-1β, and IL-6 impair blood–brain barrier (BBB) integrity by redistributing tight junction proteins, thereby enhancing permeability and facilitating entry into the central nervous system (CNS) [14,167]. Infiltrating cytokines activate microglia and astrocytes, which release glutamate and other mediators that increase synaptic excitability and contribute to central sensitization [12]. TNF-α, entering via permeable BBB or active transport, activates dorsal horn microglia, leading to increased neuronal excitability while suppressing GABAergic inhibition [14,168]. IL-1β accesses the CNS through meningeal lymphatics and binds to IL-1 receptor 1 (IL-1R1) on astrocytes, resulting in ATP release. This activates P2X4 receptors and promotes phosphorylation of NMDA receptor subunit NR2B, thereby inducing long-term potentiation (LTP) of C-fiber responses in neuropathic pain [14,169]. IL-6 penetrates via choroid plexus and stimulates astrocytic Janus kinase 2 (JAK2)-STAT3 signaling, upregulating cyclooxygenase-2 (COX-2) and prostaglandin E2 (PGE2). This downregulates neuronal potassium-chloride cotransporter 2 (KCC2), causing chloride accumulation and GABAergic disinhibition in neuropathy models [14,105]. Activated glia (e.g., microglia and astrocytes) also produce pro-inflammatory cytokines such as TNF-α and IL-1β, or chemokines like CXCL1, resulting in enhanced glutamatergic neurotransmission and excitability, reduced GABAergic neurotransmission, or both [12,170]. These effects perpetuate neuroinflammation, promote central sensitization, and contribute to pain hypersensitivity.

## 4. Microbiome Diversity in Chronic Pain

Microbial diversity is commonly described using alpha and beta diversity indices. Alpha diversity reflects the richness and evenness of microbial taxa within an individual, typically measured by Shannon, Simpson, or Faith’s phylogenetic diversity [13,27]. Beta diversity refers to differences in microbial composition between individuals or groups [13,27]. However, it is important to note that there is no universal ‘healthy’ microbiota profile, as composition varies significantly by geography, culture, and diet [13].

In chronic pain research, reductions in alpha diversity, particularly in Shannon and Faith’s indices, have been reported [13]. Similar findings have been reported in other neurological and neuroinflammatory disorders, suggesting that reduced microbial diversity may promote a pro-inflammatory environment relevant to central sensitization [13]. Beyond overall diversity shifts, phylum- and species-level alterations are consistently observed across chronic pain conditions [13,171,172,173,174]. Table 1 summarizes key species reported in fibromyalgia, migraine, osteoarthritis (OA), RA, and bladder pain syndrome [13,172,173,174]. Within the Firmicutes, members of the Clostridia class, such as *Faecalibacterium prausnitzii*, *Roseburia*, and *Coprococcus*, key SCFA producers, are reduced in chronic pain [13]. *Faecalibacterium prausnitzii*, a dominant butyrate producer, contributes to suppression of NF-κB/MAPK signaling, reduction in pro-inflammatory cytokine release, and maintenance of epithelial barrier integrity through butyrate-mediated HDAC inhibition and GPR41/43 activation [148]. Its reduction, reported in fibromyalgia, migraine, ME/CFS, and RA, may lower butyrate availability [12,13], weaken HDAC/GPR signaling, and promote neuroimmune activation and sensitization (Table 1) [14]. *Roseburia* and *Coprococcus* species, which also produce butyrate and support Treg/IL-10 signaling [14,67], are reduced in fibromyalgia, migraine, and ME/CFS [13]. Their loss could lower SCFA availability and regulatory balance, potentially favoring Th17-related inflammation [175,176]. Other SCFA producers, including *Odoribacter splanchnicus* and *Blautia obeum* (formerly known as *Ruminococcus obeum*), contribute to barrier integrity and immune regulation through SCFA production. *O. splanchnicus*, decreased in bladder pain syndrome, migraine, ME/CFS, and IBS, may provide epithelial support via SCFA-mediated mechanisms and may exert context-dependent pro-/anti-inflammatory effects [177,178]. *Blautia obeum* (formerly known as *Ruminococcus obeum*), reduced in migraine and ME/CFS, ferments carbohydrates to produce acetate and propionate [179,180,181,182,183], potentially supporting epithelial and immune function and participating in bile acid metabolism [184] (Table 1). Conversely, some Firmicutes increase chronic pain. *Clostridium symbiosum* and *Clostridium asparagiforme*, involved in bile acid metabolism [185,186], are enriched in migraine and ME/CFS [13], shifting bile acid pools toward pro-nociceptive signaling via Farnesoid X Receptor (FXR), Takeda G-Protein-Coupled Receptor 5 (TGR5) [186], and TRP channels [14,185], although reductions have been reported in OA [174] (Table 1). *Lactobacillus* species, which normally produce SCFAs and lactic acid, may promote IL-10, protect the intestinal barrier via lactate [187], convert glutamate to GABA, suppress nociceptive excitation [188], and reduce pro-inflammatory cytokines [14,148]. They are reduced in fibromyalgia and migraine, potentially altering anti-inflammatory effects and increasing nociceptor excitability. Within the Actinobacteria, *Bifidobacterium* species are reduced in fibromyalgia, migraine, and OA [13,174]. They convert glutamate into GABA [16], produce acetate [57,61], and promote anti-inflammatory cytokines; when reduced, GABA levels may drop, anti-inflammatory balance may be disturbed, and excitability may rise. In contrast, *Eggerthella lenta* is increased in migraine and ME/CFS [13], which may be linked to mucosal and systemic inflammation (Th17/IFN-γ activation) [189,190]. The *Bacteroides* species produce LPS and contribute to bile acid metabolism [72,73], are increased in ME/CFS and OA but reduced in migraine, potentially elevating LPS and activating TLR4–NF-κB signaling that promotes systemic cytokine release. It is important to note that while these altered microbiota are reported across cohorts, the evidence comes largely from cross-sectional studies; thus, it remains unclear whether this dysbiosis functions as a primary driver of pathology or represents a secondary consequence of pain-associated factors, such as altered diet, medication use, reduced physical activity, and comorbidities. Longitudinal studies are therefore needed to determine whether these microbiome changes precede or follow the onset of chronic pain. Nevertheless, available evidence suggests that many patients with chronic pain present an altered microbiome, potentially perpetuating central sensitization and the pain chronicity. Perpetuating factors are important, or even the main treatment targets, for people with chronic pain.

Metabolite-level findings complement these taxonomic shifts in chronic pain. In pediatric migraine cohorts, analysis of plasma tryptophan metabolites reveals reduced kynurenic acid alongside increased serotonin and quinolinic acid, while urinary indican is elevated as a marker of metabolic dysbiosis [191,192]. Furthermore, in nitroglycerin-induced migraine models, oral administration of sodium butyrate and sodium propionate attenuates hyperalgesia and restores intestinal permeability, supporting a functional role for microbial SCFAs in migraine pathology [193,194]. Similarly, in fibromyalgia, altered metabolite profiles have been reported, characterized by low plasma acetate and an elevated Kyn/Trp. Acetate was inversely associated with TNF-α and severity scores, linking microbial metabolite signals to inflammatory pain severity in humans [195]. In patients with ME/CFS, stool metabolomics indicate depleted fecal acetate, butyrate, and isovalerate, with key SCFA-producing taxa (e.g., Faecalibacterium) correlating with butyrate levels and fatigue severity, further linking taxonomic depletion to functional metabolic deficits [196,197]. In summary, chronic pain is consistently associated with a loss of SCFA-producing Firmicutes and Actinobacteria, alongside an overgrowth of pro-inflammatory species such as *Eggerthella*, *Clostridium*, and *Bacteroides*. Together, these changes might reduce protective metabolites and enhance inflammatory signaling, creating a microbial signature that may contribute to central sensitization and the persistence of pain.

Although research on the oral microbiome in chronic pain remains limited, several taxa show associations across RA [35,87], fibromyalgia [20,34], and migraine [20]. Within the Bacteroidetes, *Porphyromonas gingivalis*, a periopathogen enriched in RA [35,87,198], citrullinates host proteins, triggers TLR2/4 activation, and promotes local and systemic inflammation, priming anti-citrullinated protein antibody responses and potentially initiating RA [87]. A recent meta-analysis including 28 studies found a significant increase in the risk of RA in individuals with *Porphyromonas gingivalis* exposure [199]. In fibromyalgia, *Prevotella denticola* and *Solobacterium moorei* are increased [34]; both possess virulence traits such as LPS production, protease activity, and volatile sulfur compound generation. These mechanisms may contribute to periodontal inflammation and systemic immune activation [200] (Table 1), although species-specific evidence and links to neuroinflammation or pain remain limited and require further study. Finally, *Mycoplasma salivarium*, enriched in migraine [20], can activate innate immune pathways in epithelial and immune cells. Its overgrowth may contribute to mucosal inflammation and systemic cytokine signaling [201] (Table 1), but evidence connecting this to neuroinflammation or pain outcomes is lacking and warrants further investigation. Together, these findings support the hypothesis that oral dysbiosis may contribute to systemic inflammation, immune priming, and sensitization processes relevant to chronic pain. However, although theoretically plausible, studies examining whether oral dysbiosis creates or perpetuates such systemic inflammation, immune priming, and central sensitization in people with chronic pain are essentially lacking and represent an important research priority.

**Table 1:** Microbiota Species Associated with Chronic Pain and Chronic-Pain-Associated Disorders.

Genus/Species	Change in Chronic Pain	Level of Evidence	Role in Health	Reduction/Increase May Lead to →
*Faecalibacterium prausnitzii*	↓ in FM, migraine, ME/CFS [13], RA [87,202]	FM, Migraine, ME/CFS: Meta-analysis of Human observational studies [13]. RA: Human observational [87,202]	Produces butyrate → HDAC inhibition [203,204] + GPR41/43 activation → suppresses NF-κB/MAPK [203,205], ↑ IL-10 [206,207,208], maintains gut barrier [203,209]	Reduction → ↓ butyrate [210,211], weaker HDAC/GPR signaling, ↑ NF-κB activity, ↑ cytokines, sensitization
*Roseburia* spp.	↓ in migraine, ME/CFS [13], and ↑ in FM [13]	Migraine, ME/CFS, FM: Meta-analysis of Human observational studies [13].	Butyrate producers [212] → HDAC [204,213] + GPR signaling [214,215,216], Treg/IL-10 support [175,214], restrains Th17/inflammation [175,176] and via vagal GPR41 signaling suppresses central amygdala, a brain region involved in pain perception [216]	Reduction → ↓ SCFAs, Th17 skewing, ↑ inflammation, ↑ pain perception
*Coprococcus* spp. (incl. *C. comes*, *C. catus*)	↓ in CWP [172], ME/CFS, Migraine [13]	CWP: Human observational [172]. Migraine, ME/CFS: Meta-analysis of Human observational studies [13].	SCFA producers (Acetate and Butyrate) [212,217,218,219,220,221] regulate HDAC/GPR [204,213], support gut homeostasis [217] and may reduce depression and neuroinflammation [222].	Reduction → ↓ SCFAs, ↑ cytokines, ↑ low-grade inflammation [172,223]
*Odoribacter splanchnicus*	↓ in bladder pain, migraine, ME/CFS [13], IBS [224]	Migraine, ME/CFS, Bladder pain: Meta-analysis of Human observational studies [13]. IBS: Human observation study [224].	Produces SCFAs [225,226] → supports barrier integrity [178,227], ↓ gut inflammation [227], Pro-/anti-inflammatory effects (context-dependent) [177,178]	Reduction → ↑ permeability, Pro-/anti-inflammatory effects (context-dependent) [177,178]
*Balutia* (*Ruminococcus*) *obeum*	↓ in migraine, ME/CFS [13]	Migraine, ME/CFS: Meta-analysis of Human observational studies [13].	Carbohydrate Fermentation [179], [181] SCFA production (Acetate and Propionate) [179,180,181,182,183], potential epithelial/immune support via SCFA-mediated mechanisms (e.g., macrophage type I interferon responses) [182], Bile acid metabolism via bile salt hydrolase activity [184]	Reduction → impaired metabolism, ↑ inflammation
*Clostridium asparagiforme and Clostridium symbiosum*	↑ in migraine, ME/CFS [13], OA [174]	Migraine, ME/CFS: Meta-analysis of Human observational studies [13]. OA: Systematic review of Human and Animal studies [174].	Bile acid biotransformation [185,186] → FXR, TGR5 [186], TRP signaling, gut barrier permeability [185], systemic inflammation [185]	Overgrowth → disturbed BA pools, FXR/TGR5/TRP signaling; Reduction → ↓ BA metabolism; ↓ gut barrier permeability [185], systemic inflammation [185]
*Lactobacillus* spp.	↓ in FM [32], migraine [173]	FM: Human observational study [32]. Migraine: Systematic Review of Human observational studies [173]	Produces SCFAs/lactate [187], ↑ IL-10 [228], ↓ TNF-α/IL-6 [228], transforms glutamate into GABA [32], exerts a protective effect on the intestinal barrier through the metabolite lactate [187], and suppresses excitation of spinal afferent nociceptive neurons [188]	Reduction → ↑ pro-inflammatory cytokines [228], ↑ nociceptor excitability [188]
*Bifidobacterium* spp.	↓ in FM [32], migraine [13]	FM: Human observational study [32]. Migraine: Meta-analysis of Human observational studies [13].	Converts glutamate to GABA [32]; acetate production [229]; regulates cytokines [229,230,231], reduces pain sensitivity [232,233,234]	Reduction → ↓ GABA, weaker anti-inflammatory control, ↑ excitability
*Eggerthella lenta*	↑ in migraine [13], ME/CFS [235]	Migraine: Meta-analysis of Human observational studies [13]. ME/CFS: Meta-analysis of Human observational studies [235].	Pathobiont [236]; Bile acid biotransformation [185], linked to mucosal inflammation [189], systemic inflammation [189,237]	Overgrowth → Th17/IFN-γ activation [189], systemic inflammation [189,237]
*Bacteroides* spp.	↑ in ME/CFS [13], OA [19]; ↓ in migraine [13]	Migraine, ME/CFS: Meta-analysis of Human observational studies [13]. OA: Human observational study [19].	LPS producers [238]; bile acid metabolism [239]; activate TLR4 → NF-κB [238,240]	Overgrowth → ↑ LPS/TLR4 signaling [238,240], systemic inflammation [238,240]
**Oral Dysbiosis**				
*Porphyromonas gingivalis*	↑ in RA [87,198] Human observational studies [87,198].		Gingipains → citrullination of host proteins; MAPK/NF-kB activation [241]; immune evasion [242]	Overgrowth → local/systemic inflammation; anti-citrullinated protein antibodies priming [243]; potential RA initiation/progression [87].
*Prevotella denticola*	↑ in FM [34], RA [35]	FM: Human observational study [34] RA: Human observational study [35]	Periopathogen [34]; LPS and protease activity [244]; biofilm former [245]; promotes cytokine release	Overgrowth → may contribute to periodontal inflammation, but species-specific evidence is limited and further studies are needed.
*Solobacterium moorei*	↑ in FM [34]	FM: Human observational study [34]	Produces volatile sulfur compounds [34], oral gavage in mice disrupted intestinal barrier and activated NF-κB inflammation [246]	Overgrowth → may contribute to inflammation and systemic immune activation, but evidence on neuroinflammation or pain outcomes is lacking and requires further study.
*Mycoplasma salivarium*	↑ in migraine [20]	Migraine: Human observational study [20].	Commensal turned opportunist; activates innate immune cells and epithelial adhesion molecules [247,248]	Overgrowth → may contribute to mucosal immune activation and systemic cytokine signaling [247,248,249], but evidence on neuroinflammation or pain outcomes is lacking and requires further study.

**Abbreviations:** FM, Fibromyalgia; ME/CFS, Myalgic Encephalomyelitis/Chronic Fatigue Syndrome; RA, Rheumatoid Arthritis; OA, Osteoarthritis; CWP, Chronic Widespread Pain; SCFAs, Short-Chain Fatty Acids; HDAC, Histone Deacetylase; GPR, G-Protein-Coupled Receptor; NF-κB, Nuclear Factor kappa-light-chain-enhancer of activated B cells; MAPK, Mitogen-Activated Protein Kinase; IL, Interleukin; TNF-α, Tumor Necrosis Factor-alpha; Treg, Regulatory T cell; Th17, T helper 17 cell; CNS, Central Nervous System; BA, Bile Acids; FXR, Farnesoid X Receptor; TGR5, Takeda G-Protein-Coupled Receptor 5; TRP, Transient Receptor Potential channels; GABA, Gamma-Aminobutyric Acid; LPS, Lipopolysaccharides; TLR4, Toll-Like Receptor 4; TLR2, Toll-Like Receptor 2; ACPA, Anti-Citrullinated Protein Antibodies. Arrows indicate direction and proposed mechanistic links:↓ decrease; ↑ increase; → indicates a proposed mechanistic link (i.e., “leads to/results in”).

## 5. Beyond Correlation: The Elusive Path of Causation

Although alterations in the gut and oral microbiota are frequently linked with chronic pain, establishing a causal relationship remains a major challenge. It is unclear whether such microbial changes actively contribute to the onset and persistence of chronic pain or arise as consequences of pain and its related factors. Observational studies are often confounded by influences such as diet, medication use, and reverse causation, where pain itself may reshape the microbiome through lifestyle or stress-related pathways. To address these limitations, approaches such as Mendelian randomization (MR), which leverages genetic variants as instrumental variables to infer causality while minimizing confounding, and fecal microbiota transplantation (FMT) in animal models have been employed, providing stronger evidence for causality. However, microbiome MR estimates require cautious interpretation because host genetic effects on microbial traits can be complex and pleiotropic, potentially biasing “causal” signals [250].

For instance, a recent MR study [251] identified significant causal associations between 13 gut microbial taxa and chronic pain phenotypes, with eight protective and seven increasing risk. *Odoribacter* was associated with reduced neck/shoulder pain via microstructural integrity in the brain’s fornix and stria terminalis, regions involved in cognitive and emotional regulation within the corticolimbic system [251]. Similar MR approaches have revealed causal links between gut microbiota and specific conditions, such as low back pain [252], fibromyalgia [223], neuropathic pain [253], and chronic regional pain [254]. Further MR analyses extended these insights, identifying causal links between gut microbiota and multiple pain sites, including the back, knee, and abdomen [232], as well as interstitial cystitis/bladder pain syndrome [255] and chronic prostatitis/chronic pelvic pain syndrome [256].

Complementing these human genetic insights, preclinical animal FMT studies provide direct mechanistic evidence of causality. FMT from fibromyalgia patients to germ-free mice induced pain hypersensitivity, fatigue, and cognitive deficits, which were reversed by FMT from healthy donors, implicating dysbiosis in central sensitization and glial activation [120]. Similarly, in rodents, FMT from donors exposed to chronic unpredictable mild stress induced anxiety and depression-like behaviors, often comorbid with pain, and was accompanied by hippocampal neuroinflammation and altered neurotransmitter levels [257]. In neuropathic pain models, FMT containing healthy microbiota attenuated mechanical hypersensitivity, restored microbial composition, and reduced glial activation and inflammatory markers [258]. In another model, FMT from ovariectomized mice exhibiting allodynia induced allodynia in healthy mice, whereas transplantation from sham mice alleviated allodynia in ovariectomized recipients, linking microbial shifts to spinal glial activation and estrogen-modulated pain pathways [259]. A recent systematic review confirmed these findings, showing robust effects of FMT on pain-related behaviors, with meta-analyses reporting large impacts on mood, cognition, and motor function, thereby supporting the microbiota’s causal role in modulating the gut–brain axis [260].

In contrast to these robust preclinical findings, evidence for FMT in human chronic pain remains promising but preliminary. A pilot study in treatment-resistant fibromyalgia reported symptom relief, including pain reduction, following FMT from healthy donors [261]. A systematic review further suggested that FMT may reduce pain intensity and improve fatigue and quality of life, particularly in patients with fibromyalgia and irritable bowel syndrome [262]. However, the generalizability of these limited findings to other distinct pain phenotypes remains unclear. Collectively, these findings support a bidirectional causal role for the gut microbiome in chronic pain pathogenesis, potentially mediated by microbial metabolites and immune-neural crosstalk, and highlight the therapeutic potential of microbiome-targeted strategies. Further well-powered, randomized, controlled trials across multiple chronic pain conditions are needed to establish efficacy and safety. Incorporating multi-omics endpoints (e.g., metagenomics, metabolomics, and inflammatory markers) and stratifying patients based on their baseline microbiome profiles may help identify responders and improve reproducibility.

## 6. Therapeutic Opportunities

Building on this causal framework, the gut microbiome appears to contribute to the pathogenesis of chronic pain, opening the door to novel treatment modalities aimed at modulating this community. Even in the absence of causality, a possible perpetuating role of oral and/or gut microbiome in people with chronic pain would warrant therapeutic targeting of the microbiome and potentially fit into the global move towards precision pain medicine. These prospects are of particular interest to both patients and clinicians and are reinforced by anecdotal reports of symptomatic improvement following the adoption of certain lifestyle changes [263]. Contemporary evidence shows that lifestyle factors such as physical inactivity, stress, poor sleep, unhealthy diet, and smoking are not only associated with chronic pain across age groups [264,265,266,267] but also shape gut microbiome composition [263]. In particular, physical inactivity, stress, poor sleep, and unhealthy dietary patterns are linked to reduced microbial diversity, depletion of beneficial taxa, and expansion of pro-inflammatory species [13,17,268,269,270]. Favorable changes in gut microbiome composition have been observed following lifestyle interventions, with these changes linked to improvements in pain and quality of life [263]. Beyond the gut, poor oral health has also been observed in subgroups with abdominal pain [20], low back pain [37], fibromyalgia [36], and RA [38], further underscoring the role of microbial health in chronic pain. In line with best-evidence clinical practice guidelines advocating a multimodal lifestyle approach, interventions that address lifestyle factors such as diet, physical activity, sleep, stress management, and oral health, together with microbial strategies including probiotics, prebiotics, and synbiotics, may provide synergistic benefits for people with chronic pain.

### 6.1. Lifestyle Factors as Multimodal Therapy

#### 6.1.1. Diet

**Figure 7:**
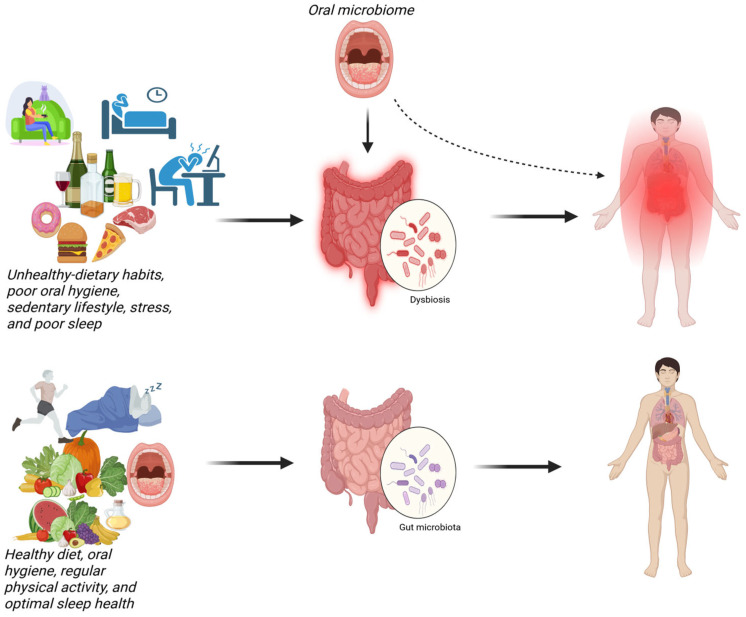
Lifestyle factors shape the oral and gut microbiota and influence pain. Created with BioRender.com.

Diet is a major determinant of gut microbiome composition and function [271], with broad implications for pain and overall health [17,263]. Diets rich in fiber from fruits, vegetables, and whole grains promote the growth of beneficial bacteria that produce SCFAs, which exert anti-inflammatory effects, strengthen the intestinal barrier, and support gastrointestinal, metabolic, and even sleep health [14,17,61,271,272]. In contrast, diets high in processed foods, sugars, and animal fats are associated with dysbiosis, reduced microbial diversity, and impaired intestinal permeability, which may contribute to disease risk [17,271,273]. Excessive intake of saturated fats is associated with unfavorable microbial shifts, whereas omega-3 polyunsaturated and monounsaturated fats (e.g., from fish oil and extra virgin olive oil) enhance microbial diversity, SCFA production, and mucosal integrity, partly via their polyphenol content [17,274]. High added sugar consumption, particularly sucrose and fructose, disrupts the microbiota, reduces SCFA production, and increases pro-inflammatory taxa, a pattern also seen in individuals with sleep disturbances [17]. Excessive consumption of red and processed meat further aggravates dysbiosis, reduces SCFA production, and promotes systemic low-grade inflammation [275]. Alcohol consumption contributes to similar microbial imbalances, weakening barrier function and amplifying permeability [17] (Figure 7). Together, these findings underscore the potential of dietary modification that emphasizes fiber, unsaturated fats, and polyphenol-rich foods while limiting processed foods, sugars, saturated fats, alcohol, and red or processed meat as a microbiome-targeted approach to mitigate chronic pain.

#### 6.1.2. Physical Activity

Physical activity is increasingly recognized as both a preventive and therapeutic strategy for chronic pain [276], with growing evidence suggesting that gut microbiota may partly mediate these benefits [277]. Regular physical activity has been shown to increase gut microbial diversity, enrich taxa involved in SCFAs metabolism, and reduce both peripheral and central sensitization [278,279]. In contrast, sedentary behavior has been linked to lower salivary microbial diversity and a higher abundance of *Streptococcus*, a genus associated with late-onset colorectal cancer, suggesting a possible connection between oral and gut microbiota in disease risk [279]. Exercise can also mitigate the harmful impact of high-fat diets by limiting inflammatory cell infiltration and preserving intestinal morphology [277], whereas high-fat diets combined with sedentary behavior induce villus enlargement via plasmacytoid and lymphocytic infiltration [280]. These changes may be counteracted by regular exercise through downregulation of COX-2 expression in both proximal and distal regions of the intestine [277]. The influence of exercise on the microbiome, however, depends on type, frequency, intensity, and duration [263]. Moderate, regular activity promotes a beneficial microbial profile, whereas irregular or excessive training can disrupt gut barrier integrity, promote bacterial translocation, and trigger dysbiosis, leading to impaired immune and gastrointestinal function [263,277]. Furthermore, activation of the HPA axis during intense physical exertion or psychological stress can further aggravate these microbial alterations [277] (Figure 7).

#### 6.1.3. Sleep and Circadian Health

Emerging evidence suggests a bidirectional relationship between sleep and the gut microbiome, mediated by the microbiota–gut–brain axis [17,281]. Sleep disruption alters microbial composition, with reductions in beneficial taxa such as *Bifidobacterium* and *Lactobacillaceae* and increases in *Firmicutes* or *Proteobacteria* [281]. These changes are accompanied by greater gut permeability and inflammation [281]. Conversely, dysbiosis can impair sleep by disrupting the production of microbial metabolites such as SCFAs and GABA [17]. Sleep restriction to 4–5 h per night for one week impairs glucose tolerance and reduces tissue insulin sensitivity [282]. Extending time in bed by one hour improves insulin sensitivity in chronically sleep-restricted healthy adults [283]. Both sleep disorders and gut dysbiosis can contribute to abnormalities in carbohydrate metabolism [17] (Figure 7). Notably, sleep disturbance is highly prevalent in chronic pain and may perpetuate dysbiosis through stress-related neuroendocrine activation, low-grade inflammation, and pain-related lifestyle changes. Therefore, optimizing sleep through structured sleep hygiene and behavioral interventions (e.g., cognitive behavioral therapy for insomnia) should be considered a key component of multimodal management to help break this vicious cycle and potentially support microbiome recovery. A balanced diet rich in plant-based foods enhances the production of sleep-regulating metabolites, potentially improving quality of sleep and benefiting overall health [17].

#### 6.1.4. Stress Management

Psychological stress may affect the oral and gut microbiomes independently, thereby forming the oral–brain axis and oral–gut axis, respectively [32,284]. The HPA axis regulates stress responses and modulates the gut–brain axis through ACTH-driven cortisol release [132,133,134]. In people with chronic pain, this axis is often dysregulated, leading to hypercortisolism or hypocortisolism. While acute cortisol has anti-inflammatory effects, chronic elevation alters microbiota [27], increases gut permeability [143], and promotes neuroinflammation [144]. Meditation therapy can improve gut microbiota composition and is associated with reduced risk of anxiety, depression and cardiovascular disease and could enhance immune function [285]. A randomized controlled trial showed that cognitive behavioral therapy for irritable bowel syndrome reduced symptom severity, with treatment success associated with baseline gut microbiome composition and concurrent post-treatment changes in both brain networks and microbiota [286].

#### 6.1.5. Oral Health

Poor oral health/oral dysbiosis triggers oral–gut inflammation, resulting in dysbiosis and compromised barrier integrity. This dysbiosis elevates intestinal permeability, allowing microbial metabolites to leak into circulation and drive pro-inflammatory cytokine production, thereby fostering systemic inflammation [40]. Oral microbiota also cross mucosal barriers during routine activities, entering the bloodstream and disseminating to organs, where they induce immune activation and chronic low-grade inflammation [109]. Recent studies suggest that the oral–gut axis may serve as a potential causal link between oral health and systemic disease [46,98,99,100] and chronic pain [37,287]. Improving oral health may downregulate inflammation through host modulation therapies, as prolonged systemic antibiotic use is discouraged owing to resistance concerns as well as devastating effects on the microbiome; instead, probiotics and bioactive metabolites have been extensively explored for modulating inflammation [288].

Multimodal treatments combining nutritional intervention, oral hygiene, physical activity promotion, and sleep and stress management work synergistically with microbial or pharmacological therapies, ensuring that beneficial species can colonize and function in a supportive host environment.

## 7. Current Challenges and Future Directions

The gut microbiome has emerged as a promising modifiable factor, influenced by lifestyle factors such as diet, sleep, physical activity, stress regulation, and oral hygiene, as well as microbial interventions including probiotics, prebiotics, and synbiotics. These factors position it as a potential therapeutic target for chronic pain conditions such as fibromyalgia, OA, migraine, irritable bowel syndrome and low back pain. However, evidence-based recommendations are limited due to their high variability; no universal “healthy” profile exists, as composition varies by geography, culture, and diet (e.g., European cohorts dominated by *Firmicutes* and *Bacteroidota*, versus diverse Asian populations with *Prevotella*, *Faecalibacterium*, and *Lactobacillus*) [13]. The complexity of this ecosystem warrants careful consideration before attempting to “normalize” the abundance of disrupted bacterial species in chronic pain. For instance, *Prevotella copri* is depleted in fibromyalgia yet elevated in inflammatory arthritis, highlighting the need for caution when intervening in its population [289]. Similarly, *Roseburia* spp. show reduced abundance in migraine and ME/CFS but are elevated in fibromyalgia [13], while *Bacteroides* spp. are increased in ME/CFS and OA [13,19] but decreased in migraine [13]. These context-dependent roles suggest that specific taxa cannot be universally categorized as beneficial or harmful. Therefore, moving toward precision medicine requires future research that prioritizes condition- or comorbidity-specific microbiome profiling and functional analyses to identify causal pathways before firm recommendations can be made. Despite these challenges, recent advancements in metabolomics and microbiome research provide an opportunity to move beyond descriptive associations and toward mechanistic insights. First longitudinal cohort studies with repeated microbial, metabolic, and immune profiling are needed to determine whether microbiota changes precede or follow the onset of chronic pain, with frameworks such as directed acyclic graphs applied to strengthen causal inference. Second, multi-omics approaches (metagenomics, metabolomics, transcriptomics, immunophenotyping) should be applied to identify causal pathways linking microbial metabolites (e.g., SCFAs, LPS, bile acids) to neuroimmune activation and central sensitization in people with chronic pain. For instance, combining gut microbiome profiling with serum metabolomics (including SCFAs) and circulating cytokine analysis allows researchers to test whether specific metabolite deficits co-vary with inflammatory signatures, as recently illustrated in a multi-omics analysis of samples obtained from patients with fibromyalgia [32]. Third, preclinical studies of FMT have shown promise in modulating pain-related behaviors, suggesting translational potential. However, human application requires careful evaluation of safety, durability, and specificity of microbial transfer, particularly given the heterogeneity of donor microbiomes and risk of unintended effects. Finally, precision pain medicine approaches are needed. Microbiome composition varies substantially across geography, diet, and cultural practices, suggesting that personalized or stratified interventions based on baseline microbial and metabolic profiles will be necessary rather than assuming a universal therapeutic strategy. The latter not only applies to direct therapeutic targeting of the microbiome, such as done during FMT, probiotics, prebiotics, and synbiotics, but also to indirect strategies such as dietary interventions.

## 8. Conclusions

This narrative review highlights the interplay between oral and gut microbiota, neuroinflammation, and central sensitization in the pathogenesis of chronic pain. Emerging evidence from microbiome research shows alterations in oral and gut microbial communities in chronic pain populations, particularly a reduction in SCFAs-producing taxa (e.g., *Faecalibacterium prausnitzii*, *Roseburia* spp., *Bifidobacterium* spp.) and an overrepresentation of pro-inflammatory species (e.g., *Bacteroides* spp., *Eggerthella lenta*). These compositional shifts are accompanied by altered microbial metabolites, including SCFAs, bile acids, tryptophan derivatives, and cell wall components, which modulate gut–brain communication, intensify neuroinflammation, and promote both peripheral and central sensitization through immune, neural, and endocrine pathways.

While findings from animal models, MR, and early FMT studies suggest a potential causal role of dysbiosis, clinical translation remains incomplete, as human data are still preliminary and confounded by lifestyle, diet, and comorbidities. The complexity and variability of the microbiome underscore the need for rigorous longitudinal, mechanistic, and stratified research. Future work should integrate multi-omics approaches and apply robust causal inference to establish whether microbial metabolites are causal drivers of sensitization. Lifestyle factors such as diet, sleep, physical activity, and stress warrant particular attention, since they shape microbial composition and pain trajectories. Research should evaluate whether modifying these factors can beneficially alter the microbiome. Future strategies should combine dietary, lifestyle, and microbial interventions tailored to individual microbiota profiles to effectively address the heterogeneity of chronic pain.

## Data Availability

No new data were created or analyzed in this study. Data sharing is not applicable to this article.

## Abbreviations

The following abbreviations are used in this manuscript:

Acetyl-CoA	Acetyl coenzyme A
LPS	Lipopolysaccharides
MAMPs	Microbial-associated molecular patterns
TLR	Toll-like receptors
CRP	C-reactive protein
NF-κB	Nuclear factor kappa-B
TNF-α	Tumor necrosis factor-alpha
IL	Interleukin
GPR	G protein-coupled receptor
FM	Fibromyalgia
SCFA	Short-chain fatty acid
FMT	Fecal microbiota transplantation
ME/CFS	Myalgic Encephalomyelitis/Chronic Fatigue Syndrome
OA	Osteoarthritis
CWP	Chronic Widespread Pain
GABA	Gamma-Aminobutyric Acid
BA	Bile acids
FXR	Farnesoid X Receptor
TGR5	Takeda G-Protein-Coupled Receptor 5
NMDA	N-methyl-D-aspartate
JAK2	Janus kinase 2
STAT3	Signal transducer and activator of transcription 3
COX2	Cyclooxygenase-2
PGE2	Prostaglandin E2
CCL2	C–C motif chemokine ligand 2
CXCL1	C–X–C motif chemokine ligand 1
Ahr	Aryl hydrocarbon receptor
ACTH	Adrenocorticotropic hormone
CRH	Corticotrophin-releasing hormone
NLRs	Nucleotide-binding oligomerization domain-like receptors
PRRs	Pattern recognition receptors
BBB	Blood–brain barrier
HDAC	Histone deacetylase
RA	Rheumatoid arthritis
PAMPs	Pathogen-associated molecular patterns
MAPK	Mitogen-activated protein kinase
TRP	Transient receptor potential
MR	Mendelian randomization
HPA	Hypothalamic–pituitary–adrenal
TRPA1	TRP ankyrin 1
